# DeepSort: deep convolutional networks for sorting haploid maize seeds

**DOI:** 10.1186/s12859-018-2267-2

**Published:** 2018-08-13

**Authors:** Balaji Veeramani, John W. Raymond, Pritam Chanda

**Affiliations:** Dow AgroSciences LLC, 9330 Zionsville Rd, Indianapolis, 46268 IN USA

**Keywords:** Corn, Double haploid induction, Agriculture, Convolutional neural networks, Molecular markers, Deep learning

## Abstract

**Background:**

Maize is a leading crop in the modern agricultural industry that accounts for more than 40% grain production worldwide. THe double haploid technique that uses fewer breeding generations for generating a maize line has accelerated the pace of development of superior commercial seed varieties and has been transforming the agricultural industry. In this technique the chromosomes of the haploid seeds are doubled and taken forward in the process while the diploids marked for elimination. Traditionally, selective visual expression of a molecular marker within the embryo region of a maize seed has been used to manually discriminate diploids from haploids. Large scale production of inbred maize lines within the agricultural industry would benefit from the development of computer vision methods for this discriminatory task. However the variability in the phenotypic expression of the molecular marker system and the heterogeneity arising out of the maize genotypes and image acquisition have been an enduring challenge towards such efforts.

**Results:**

In this work, we propose a novel application of a deep convolutional network (DeepSort) for the sorting of haploid seeds in these realistic settings. Our proposed approach outperforms existing state-of-the-art machine learning classifiers that uses features based on color, texture and morphology. We demonstrate the network derives features that can discriminate the embryo regions using the activations of the neurons in the convolutional layers. Our experiments with different architectures show that the performance decreases with the decrease in the depth of the layers.

**Conclusion:**

Our proposed method DeepSort based on the convolutional network is robust to the variation in the phenotypic expression, shape of the corn seeds, and the embryo pose with respect to the camera. In the era of modern digital agriculture, deep learning and convolutional networks will continue to play an important role in advancing research and product development within the agricultural industry.

## Background

Feeding the growing population amidst shrinking farm lands across the world requires increases in innovation and efficiencies in agricultural output through high yielding crops. Maize (aka corn) is a major crop that accounted for 28.3% harvested acres within US in 2015, with 38,105 million bushels produced across the world in 2015-2016 [1]. Maize breeding programs generate better yielding and more predictable hybrid seed varieties by crossing two distinct inbred lines. Traditional development of inbred lines, whose copies of genomes are 99% identical, takes about 6-7 generations of recurrent selfing or crossing. Double haploid (DH) based induction technology enables large commercial breeding programs in Europe, North America and China to efficiently generate homozygous lines within 2-3 generations of breeding [2]. For example, using DH process, Dupont-Pioneer has reported that they have developed a greater number of inbred lines since 2012 than they had produced in the first 80 years of their breeding program. [3]. In addition to the shortening of the generation time, DH process provides other benefits such as simplified logistics, efficiency and precision of selection, accelerated product development, and fulfillment of DUS (distinctness, uniformity, and stability) requirements for plant variety protection [2].

Sorting the diploids from the haploids seeds is a critical step in a DH induction process. Double haploid based induction uses a dominant anthocyanin color marker or gene, referred to as R1-Navajo (R1-*nj*), to distinguish putative haploid seeds from diploids. This R1-*nj* marker when expressed in a particular tissue leads to its purple coloration. R1-*nj* marker is expressed in both the outermost layer of the maize endosperm (aleurone) as well as the embryo (scutellum) in diploid maize seeds, whereas in haploid seeds it is expressed only in the endosperm layer. Using these visual differences, the most common method for sorting haploid seed is by a manual inspection process [4, 5]. In an agricultural industry setting, hundreds of thousands of seeds are sorted to separate haploids from the diploids during every breeding cycle. Manually sorting these high volumes of haploid/diploid seeds by visual discrimination is both labor intensive and error prone, and developing automated methods to classify haploids from diploids is critical.

To our knowledge, within the agricultural industry there have been two automation efforts using the R1-*nj* molecular marker system [6, 7]. The primary advancement of the disclosure [6] can be attributed to developments in the mechanical handling and image acquisition; however the image analysis methods are preliminary (uses thresholding with erosion and fill morphological operations) and its performance demonstrated in only 24 seeds. In the patent disclosure [7], a different mechanical system along with a PC Eyebot system (Sightech Vision Systems, Inc.) was implemented for image classification that recovered 38-53% haploids and 92-98% diploids correctly. However robust approaches in recovering haploids at industrial scale are lacking.

Automated sorting of haploid seeds from the diploid seeds robustly is challenging due to the differences in the expression of the R1-*nj* marker in maize seeds from different genetic backgrounds used in DH induction [8, 9], morphological variations due to incomplete pollination of corn cobs, and several environmental factors [10, 11]. In addition, variations that naturally arise during real-time image acquisition in field settings pose a challenge to the image analysis methods. Researchers have resorted to developing alternative phenotyping methods based on oil content [9] and NMR [5]. However, the throughput of these methods are constrained by multiple image acquisitions [9], and requires expensive equipment as compared with visible range RGB cameras. In this context, development of robust computer vision methods to enable automated seed sorting becomes imperative.

Recent developments in deep learning algorithms and availability of modern powerful GPUs have spurred a revolution in the areas of image classification, speech recognition, and genomics [12]. State-of-the-art performance was demonstrated in the ImageNet challenge where the images were classified into a thousand categories [13] using deep convolutional networks. Convolutional neural networks (CNN) were initially developed to efficiently represent images using neural nets with far fewer parameters (using local connectivity and weight sharing) and train them using backpropagation algorithm [14]. CNN has been widely used for several applications such as predicting sequence specificities for DNA and RNA binding proteins [15], chromatin effects of sequence alternations [16], self driving vehicles, automated phenotyping of developing *C. elegans* embryos and connectomics [12].

Applications of convolutional networks are starting to emerge in agriculture/plant sciences recently. Convolutional networks have been used to recognize paddy field pests localized using saliency maps [17], identify 44 different plant species using leaf images collected at the Royal Botanic Gardens, Kew, England [18] and classification of forest and agricultural regions in Indian Pines hyperspectral image dataset [19].

In this work, we propose an application of a convolutional network (DeepSort) to discriminate maize haploids from diploids using several thousand corn seed images based on our earlier preliminary work [20]. We demonstrate that performance of the convolutional network closely matches the visual classification of the seeds by the human experts. We also show this performance remains robust under diverse lighting conditions, seed shapes, embryo orientation relative to the camera field of view, and heterogeneous genetic backgrounds; something that has remained challenging in practice. Using visualizations, we show that the convolutional network derives information that are discriminative of haploids and extracts features from the embryo regions. Our experiments using multiple network architectures indicate that the presence of more layers (i.e. deeper network) contributes to improved classification accuracy.

## Methods

### Diploid and haploid images

**Fig. 1:**
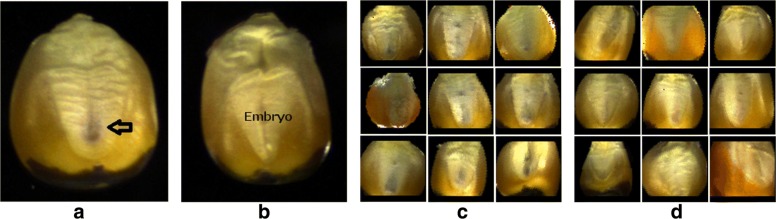
Corn seeds expressing the R1-*nj* marker in (**a**) Diploid and (**b**) Haploid seeds. The marker is only expressed in the diploid embryo as a vertical dark purple patch indicated by the arrow in (**a**). Variability in visual indications of marker expression, seed morphology, color and texture, embryo positioning with respect to camera, and lighting conditions across multiple (**c**) diploid and (**d**) haploid seeds

Corn seeds expressing the R1-*nj* marker are shown in the Fig. 1. Haploid seed embryos that receive only the maternal genetic material do not show the purple coloration from dominant R1-*nj* marker expression (see Fig. 1b in the region marked embryo), but diploid seed embryos that also receive the genetic material from the inducer carrying the R1-*nj* marker exhibits purple coloration in the embryo (shown next to the arrow in Fig. 1a). R1-*nj* marker expressed in the endosperm of both diploid and haploid seeds leads to a dark coloration as seen at the bottom of the seed images shown in Figs. 1a & 1b (below the embryo). We acquired images of 4021 seeds for training, and 710 seeds for testing the performance of the automatic seed classification system. A substantial heterogeneity in the level of expression of the R1-nj marker, seed morphology, color and embryo region texture is observed in our dataset. In addition, image acquisition introduces further variations such as lighting inconsistencies and embryo positioning relative to the camera field of view. Sample images from our dataset that demonstrate this heterogeneity in both diploids and haploids are shown in Figs. 1c & 1d respectively.

Our dataset consists of 4731 RGB images of corn seeds (3779 diploids, 952 haploids) obtained from multiple proprietary inbred lines. Seeds were manually classified into haploid and diploid categories using high resolution images by a human expert. The images were acquired under realistic settings across several days and different genetic populations using uEye high-speed cameras. The image acquisition apparatus returns two images; approximately half the images do not depict the embryo side and were discarded. The raw 640 × 480 pixel images were pre-processed using basic techniques (cropping, centering and resizing). Each seed was repositioned so that it’s pixel-based center of mass was located in the middle of 64 × 64 cropped image and having its tip oriented upward. These 64 × 64 RGB images were used for training the convolutional network and testing its performance.

### Convolutional network

**Fig. 2:**
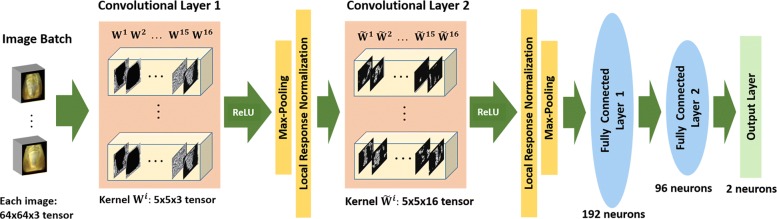
Schematic architecture of DeepSort Convolution network “Arch-1” used for classifying maize seeds. Input maize images are convolved with 16 filter kernels in the first convolutional layer followed by pooling and normalization layers. Outputs of these operations are again convolved with 16 kernels in the second convolutional followed by pooling, normalization and two fully connected layers

We adopt an exemplar CNN architecture for the classification of haploid and diploid seeds shown in Fig. 2 (termed DeepSort). It comprises of the following layers: two convolutional layers each followed by max-pooling and local-response-normalization layers, two densely connected layers and an output layer. We first describe each of these components briefly.

We start with description of the convolutional layer. Formally, let the input to a convolutional layer be represented by the tensor $\mathbf {X} \in \mathbb {R}^{N \times M \times C}$. Let a convolutional layer be comprised of *D* kernels. Each kernel (or weight) is a tensor, $\mathbf {W}^{(l)} \in \mathbb {R}^{d \times d \times C}, \{l=1\dots D\}$ that applies a 2-dimensional convolution operation on *d*×*d* patches in each of the *X*_··*k*_ plane (of dimension *N*×*M*) of the input tensor **X**, and with stride *s*. For example, if **X** represent the input image tensor, $X_{\cdot \cdot k} \in \mathbb {R}^{N \times M}, \{k=1\dots C\}$ represents a N × M pixel image across a color channel *k*, with *C*=3 such color channels (RGB). The convolution is followed by a non-linear activation function *f*(*z*)=*max*(0,*z*) (Rectifier Linear Unit or ReLU). This results in neuronal activations of the form $f\left ({\sum \nolimits }_{k=1}^{C} \left (W^{(l)} \circ X_{i:i+d-1,j:j+d-1,k}\right) + b\right) \in \mathbb {R}$. Here “ ∘” denotes the Hadamard product, $b \in \mathbb {R}$ is a bias term; *X*_*i*:*i*+*d*−1,*j*:*j*+*d*−1,*k*_ represents the *d*×*d* subregion on 2-dimensional matrix *X*_··*k*_; and $W^{(l)} = \mathbf {W}^{(l)}_{\cdot \cdot k}$, a *d*×*d* slice of the tensor. Convolutions over the entire input tensor **X** using stride size *s* and padding *p* produces activation matrix $A_{l} \in \mathbb {R}^{\left (\frac {N-d+2\cdot p}{s}+1\right) \times \left (\frac {M-d+2\cdot p}{s}+1\right)}$, which represents the activations of all the neurons in a slice *l*,{*l*=1…*D*} that share the weight tensor **W**^(*l*)^. Each neuron in a convolutional layer is connected only to a local region (defined by the convolution window) of the input spatial volume, but to the full depth *C* of the input tensor. Repeating this pattern for *l*=1…*D*, all the neurons located on the similar region of a plane but along the depth *D* look at the same region of the input tensor through different kernels **W**^(*l*)^. A convolutional layer is followed by max-pooling and local-response-normalization layers. The max-pooling layer downsamples the input tensor by partitioning it into a set of non-overlapping sub-regions and outputs the maximum value of each such sub-region. The normalization layer mimics lateral inhibition in real neurons and performs damping of neuronal responses that are uniformly large in a local neighborhood, while boosting neuron responses that are moderately strong in a local neighborhood of weaker responses [13].

In our primary design (called “Arch-1”, see Fig. 2), we have *D*=16 kernels in the first convolutional layer looking at RGB images (i.e tensor $\mathbf {X} \in \mathbb {R}^{N \times M \times 3}$). This is followed by max pooling and normalization layers. The first set of layers is followed by the second convolution, normalization and max pooling layers, in that order. The second convolutional layer also has *D*=16 kernels and processes as input, a tensor that is the output from the preceding normalization layer. For both our convolutional layers, *d*=5, *s*=1 and *p*=2, other kernel receptive field patch sizes (*d*=3,7) provided similar results. These layers are followed by 2 fully connected layers (with 192 and 96 neurons respectively) that are connected to all the neurons from the preceding layer, which is followed by an output layer having 2 neurons that represent the haploid and diploid classes. Our initial design is motivated by the network used for classifying cifar10 data of natural images (https://code.google.com/p/cuda-convnet/). The choice of the number of kernels for classifying haploids from diploids were four times smaller than the cifar10 network that classifies images into 10 categories (roughly 5 times more classes than our problem).

We further experimented with different architectures where the number of kernels in the convolutional layers and neurons in fully connected layers were reduced by half for every subsequent architecture. We considered two such architectures, followed by an architecture that reduced the number the layers. Briefly the architectures are : (1) “Arch-2” : 8 kernels in each of the two convolutional layers, 96 and 48 neurons in first and second fully connected layers respectively; (2) “Arch-3” : 4 kernels in each of the convolutional layers, 48 and 24 neurons in first and second fully connected layers respectively; and (3) “Arch-4” : a single convolutional layer with 4 kernels and a fully connected layer with 24 neurons. In all architectures, each convolutional layer is followed by max-pooling and local-response-normalization layers similar to “Arch-1”.

Convolutional networks were implemented with the library tensorflow version 0.8.0 (https://www.tensorflow.org/) and K80 Nvidia GPUs. During training, parameter values (such initial learning rate (0.1), learning rate decay factor (0.1), number of epochs per decay(350), moving average decay (0.999)), images transformations (approximate whitening), and data augmentation distortions (random flip, image brightness and contrast distortions) were used. Furthermore, we used the strategies that have been reported to reduce model overfitting during training and improve performances of convolutional networks [13], such as data augmentation by introducing transformations to the training data, and moving average weight decay. We used a batch size of 64 samples, and training was carried out for 400,000 iterations ensuring the algorithm was not trapped in local optima with unfavorable classification accuracy.

## Results and discussion

### Classification performance of deep convolutional network

We use 4021 randomly chosen images of corn seeds (809 haploids and 3212 diploids) for training and 710 images (143 haploids and 567 diploids) for testing. The training and test datasets contained 20% haploids. The training dataset was further split in 5-folds to assess the performance of different network architectures (discussed in “Architectures of deep convolutional networks” section).

We also compared the performance of the convolutional network with an image analysis pipeline that uses feature extraction followed by classification, an approach similar to one used by [21] to classify pepper seed images. We extracted Haralick texture features [22], local binary patterns, zernike moments, and shape features using MATLAB ‘regionprops’ (total 84 features).

Using these features we experimented with several classifiers: Support Vector Machine (SVM), Random Forest (RF), and Logistic Regression (LR) [23]. In our experiments, Haralick texture features were found to better discriminate haploids from diploids as compared to morphology, color and shape features. Therefore we also report the performances of the classifiers using only 13 Haralick features in addition to using all the features (see Table 1). Image handling and feature extraction were performed using the Mahotas 1.4 library package [24] and MATLAB, and the classifiers was implemented using scikit-learn library [25]. For each classifier, the respective parameters in scikit-learn terminology (*gamma* and *C* for SVM with a radial basis kernel; *n*_*estimators, min*_*samples*_*split, min*_*samples*_*leaf* for RF; regularization parameter *C* for LR), were chosen using a grid search with 5-fold cross-validation (CV).

**Table 1:** Comparison of classification accuracies of DeepSort and other classifiers. Other classifiers were tested with all features described in text (values within brackets), and using only Haralick texture features (values outside brackets). CV indicates 5-fold cross-validation

	DeepSort	Random Forest	SVM	Logistic Reg
CV	0.961	0.840 (0.823)	0.857 (0.836)	0.749 (0.777)
Train	1.000	1.000 (0.997)	0.911 (0.994)	0.751 (0.786)
Test	0.968	0.845 (0.824)	0.876 (0.839)	0.775 (0.772)

The performance results are summarized in Table 1. We observe DeepSort is able to classify the training images perfectly which shows enough flexibility in its architecture to learn the heterogeneity of the samples in training dataset. In our experiments using the test dataset, we observe DeepSort outperforms all other machine learning classifiers, and attained a classification accuracy of 0.968. Among the other methods compared, SVM achieved the highest classification accuracy (0.876) using Haralick features; 9.2*%* lesser than DeepSort. We discuss only this comparitive method in the next sections. We observe the test and cross-validation accuracies of compared methods to be lacking even though their training accuracies were high (RF Haralick, all features; SVM all features), demonstrating their inability to generalize. Further, we also observed the SVM classifier to have a large number of support vectors (1390 with full training dataset and Haralick features).

We examined the confusion matrix of DeepSort and the best performing machine learning classifier (SVM with Haralick features) to understand the performance of these methods on each category individually using the test data set (See Table 2). Diagonal values of the confusion matrix represent the correct classification of haploids and diploids into their appropriate categories, and off-diagonals represent mis-classifications. DeepSort misclassified 11 diploid images as haploids, and 12 haploid images as diploids, while SVM misclassified 22 diploid images as haploids and 66 haploid images as diploids. We observe SVM is biased towards classifying haploid images as diploids, possibly representing the distribution of the diploid images in the dataset. Our attempts to compensate for this class bias in the SVM (using ‘balanced’ setting for class_weight parameter in Scikit-learn) decreased the training error but led to a larger cross-validation error (results not shown).

**Table 2:** Confusion Matrix for DeepSort and SVM

	DeepSort		SVM	
	Pred-Diploid	Pred-Haploid	Pred-Diploid	Pred-Haploid
True-Diploid	556	11	545	22
True-Haploid	12	131	66	77

Pred: Predicted label; True: Actual label; Using test data (143 haploids, 567 diploids)

### Visualizing the convolutional network neurons

We conducted visualizations of the kernels and neuronal activations of the two convolutional layers in order to understand the features learnt by the network in achieving superior classification performance. Understanding the features learned by deep neural networks, and how these features are effectively combined towards superior classification is complex (as these networks often have several hundreds of thousands of parameters), and is an active field of ongoing research [26, 27]. Visualization of the neuronal activations in a convolution layer, weight tensors and bias parameters; image regions/features that lead to maximal neuronal activations; and receptive fields of individual neurons are some of the current techniques employed towards this goal [27]. In our work, we chose to focus on visualizations of the neuronal activations of the first two convolutional layers, as the structure of the input images are somewhat preserved in these two layers. We show that these visualizations help to gain key insights into the functions performed by the convolutional layers in extracting discriminatory seed features.

**Fig. 3:**
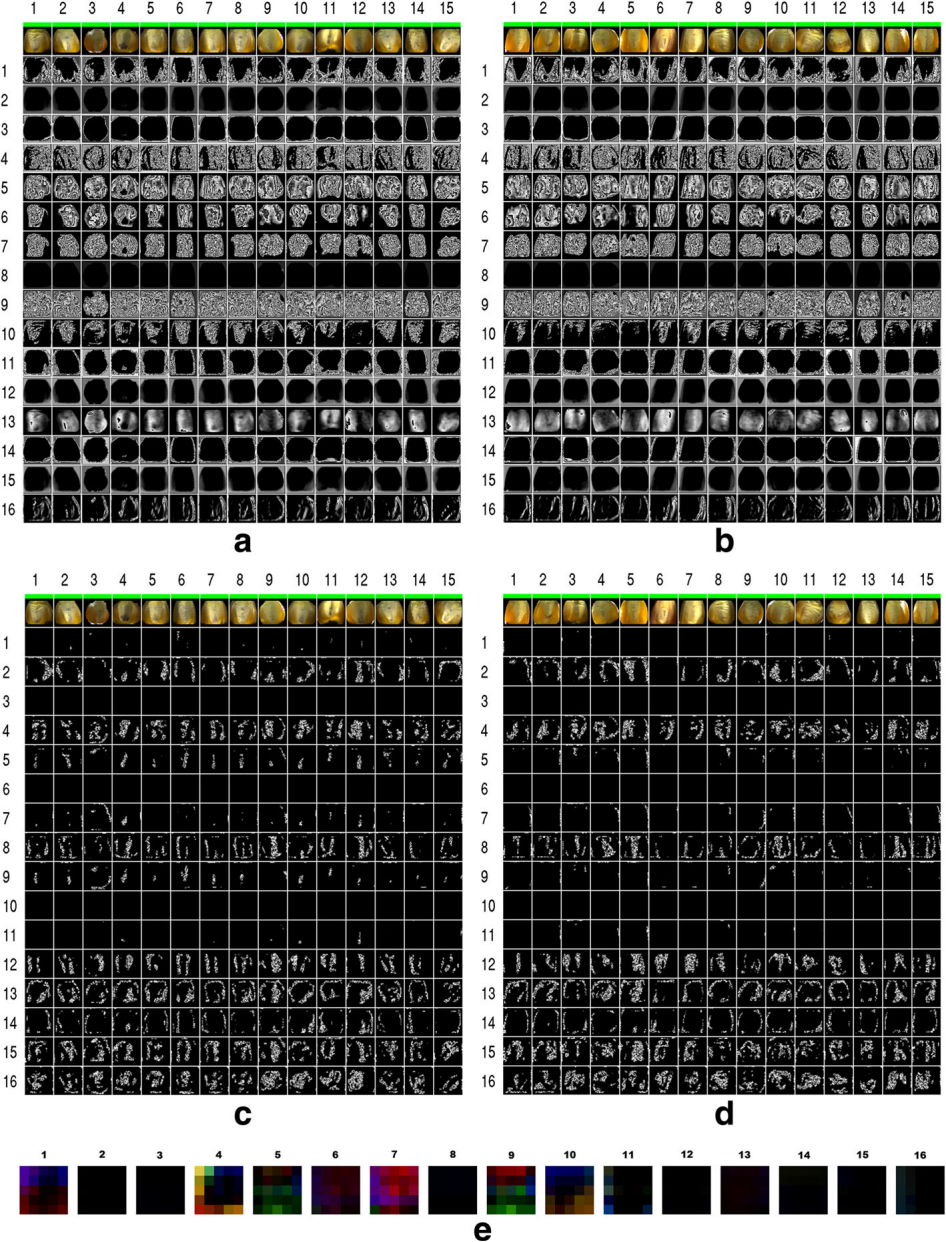
Figure (**a**, **b**) shows the activations of all neurons in the convolutional layer 1 (each row corresponds to the activations that share a kernel) across images of 15 random diploid (**a**) and haploid (**b**) seeds (each column for a seed shown at the top row) from the test data set. Similar to figures (**a**, **b**), figures (**c**, **d**) shows the activations of neurons in the convolutional layer 2 across the same set of seeds. Kernels in the convolutional layers 1 and 2 perform various feature extractions and their complex compositions. For example, kernels 3 of first layer segments the seed from the background, and kernel 5 of the second layer provides discriminatory features (for other examples see text). Figure (**e**) shows visualizations of 16 kernels from the convolutional layer 1

Figure 3 shows the activations of the neurons in the first two convolutional layers to an input of 30 randomly chosen haploid and diploid seeds from our test dataset (i.e. 15 of each category, and denoted by columns numbered 1-15). The activations of first convolutional layer neurons to diploid and haploid seeds are shown in Figs. 3a and 3b, respectively. An image(*i*,*j*) in the grid of each subfigure a-d of Fig. 3 denotes activations of all neurons that share a kernel *i* on an input from the seed image in column *j* shown at the top. Visually comparing the images in haploid and diploid categories across a row of Fig. 3 (a & b or c & d) allows one to identify kernels that are discriminatory and the operations performed by them. The 16 kernels in the first convolutional layer each looking at 5×5×3 segment of an input RGB image are shown in Fig. 3e. Kernels in the second layer, looking at an input tensor 5×5×16 with a depth larger than three, are not shown.

We observe several interesting patterns. The neurons in the first layer perform image pre-processing operations, while those in the second layer synthesize higher level features. Looking at the activations of neurons in the first convolution layer in Figs. 3a and 3b, we observe kernels 2, 3, 8, 11, 12, 14, 15 to broadly segment the seed from the background, however with differences near the seed boundaries and output intensity. We also looked at the corresponding kernel tensors of these neurons, and observe them to be different in terms of their magnitudes, patterns within a given channel, and across the different RGB channels (see Fig. 3e), possibly contributing to the robustness of performance across the heterogeneous seeds. Although some kernels are seen as dark patches in Fig. 3e, they are not uniformly zero as seen from the raw values. Several other kernels in the first convolution layer (kernels 4-7,9-10) extract features related to seed texture, while kernel 1 extracts texture in endosperm region but not in the embryo. Kernel 13 serves to perform an intensity based segmentation, while kernel 10 accentuates embryo regions is diploid seeds (and in some haploids).

The second convolutional layer kernels extract complex features that do not reflect the exact shape of the seeds or embryo features. Rather these features encode more abstract input concepts combining features extracted from the max-pooled and normalized output of the first convolutional layer. We notice that fewer neurons in the second convolutional layer are active as observed by fewer white pixels in Figs. 3c and 3d. Strikingly, activations from the kernels 5 and 9 are already discriminatory of most diploid and haploid seeds, roughly highlighting the purple dark regions in diploid seeds. Kernels 12 and to some extent 15 marks the brighter regions of the embryo in the diploid seeds with two vertical patches. Information from these activations are further nonlinearly combined by the fully connected layers to achieve robust performance.

### Architectures of deep convolutional networks

We explored the effect of the architecture on the performance of the convolutional network in classifying haploid seeds from diploids by changing the number of kernels in the convolutional layer, neurons in the fully connected layer, and depth (i.e, number of layers) of the network (see Table 3). We considered two different architectures (“Arch-2” and “Arch-3”) with the same number of layers as “Arch-1”, but with reduced number of kernels and neurons in the fully connected layer. “Arch-2” had eight kernels in the first and second convolutional layers, and “Arch-3” had four kernels in the convolutional layers (see “Convolutional network” section for the number of kernels/neurons, and Table 3 for the number of parameters in each layer). Going from “Arch-1” to “Arch-3”, the number of parameters decreases roughly four fold for each step. We also considered a shallower architecture (“Arch-4”) with a single convolutional and fully connected layer.

**Table 3:** Effect of CNN architecture on classification accuracy (cols. 2,3) and number of parameters per layer (cols. 4-9) in each architecture

Method	Train	Test	Conv1	Conv2	Full1	Full2	Output	Total
Arch-1	1.000	0.968	1216	6416	786,624	18,528	194	812,978
Arch-2	1.000	0.968	608	1608	196,704	4656	98	203,674
Arch-3	0.992	0.941	304	404	49,200	1176	50	51,134
Arch-4	0.989	0.935	304	-	98,328	-	50	98,682

Conv[1/2]: [first/second] convolution layers, Full[1/2] : [first/second] fully connected layers, output: final softmax layer

We observe that the performance of the “Arch-2” (0.968 on test set) is similar to that of “Arch-1”, whereas in “Arch-3” the accuracy is reduced by 0.027 (or roughly 15 more test images were misclassified). We also observe that the training accuracy goes down by 0.008 (roughly 32 more training images were wrongly classified). We further reduced a convolutional and a fully connected layer and obtained “Arch-4” to assess the impact of having a shallower network. The performance on test data dropped to 0.935 in “Arch-4” (a slight reduction as compared to “Arch-3”, but lesser than “Arch-2” by 0.033), even though the total number of parameters is more than “Arch-3” (since “Conv1” of “Arch-4” has more neurons than “Conv2” of “Arch-3”). Although “Arch-1” displayed similar classification accuracy as “Arch-2” (with four times more parameters than “Arch-2”), future experiments have to be performed to study if more trainable parameters enable “Arch-1” to be more robust to unseen variations and more heterogeneity.

## Conclusion

Our experiments provide evidence to the usefulness of DeepSort in discriminating haploids from diploid seeds in the double haploid induction process, and to its robustness amidst variations arising from biological factors and image acquisition. We establish such robustness using thousands of seed images obtained in an industrial scenario from different genetic backgrounds. Our visualizations indicate that embryo’s features are being extracted by the network, which may be used further to classify the seeds, as are carried out manually by agricultural field workers. We further observe that deeper architectures provide better classification accuracies as compared to shallower architectures. In the future, we intend to develop more general deep networks, which can classify haploids from diploids using two un-identified images with one showing the embryo and other not showing it, and with minimal pre-processing.

Convolutional networks and other deep learning methods, though popular in several commercial applications (e-commerce, social networking, retail, automotive, etc.), have only began to find applications within agriculture recently. The approach we use to classify corn seed images into haploid or diploid categories, could be extended to other agricultural applications. A few of these include sorting seed images of crops such as soybean, canola, etc. into various categories (e.g. high oil vs low oil, high-moisture vs low-moisture, deformed vs non-deformed, etc.), detecting pest infestations using remote sensing images, estimating plant vigor using field images from drones, assigning diseased status to plants from leaf images, insect mortality rate estimation from bioassay images, etc. The combination of different deep network architectures with a variety of sensors (such as multispectral, infra-red, MRI, etc.) offers enormous possibilities, and will contribute to next generation agricultural phenotyping. In addition, modern high-throughput technologies has enabled agricultural industries to collect large scale molecular datasets. Deep networks can be applied to several such agricultural biotech predictive applications using biological sequences (DNA, RNA, proteins etc.), genetic (SNPs other genetic variations etc.), chemical, environmental and phenotypic data.
